# Engineering Tissue-Informed Biomaterials to Advance Pulmonary Regenerative Medicine

**DOI:** 10.3389/fmed.2021.647834

**Published:** 2021-04-08

**Authors:** Donald R. Campbell, Christiana N. Senger, Amy L. Ryan, Chelsea M. Magin

**Affiliations:** ^1^Department of Bioengineering, Denver, Anschutz Medical Campus, University of Colorado, Aurora, CO, United States; ^2^Division of Pulmonary, Critical Care and Sleep Medicine, Department of Medicine, Hastings Center for Pulmonary Research, University of Southern California, Los Angeles, CA, United States; ^3^Department of Stem Cell Biology and Regenerative Medicine, University of Southern California, Los Angeles, CA, United States; ^4^Department of Pediatrics, Anschutz Medical Campus, University of Colorado, Aurora, CO, United States; ^5^Division of Pulmonary Sciences and Critical Care Medicine, Department of Medicine, Anschutz Medical Campus, University of Colorado, Aurora, CO, United States

**Keywords:** disease modeling, regenerative medicine, pulmonary, hydrogel, tissue-informed engineering, biomaterials

## Abstract

Biomaterials intentionally designed to support the expansion, differentiation, and three-dimensional (3D) culture of induced-pluripotent stem cells (iPSCs) may pave the way to cell-based therapies for chronic respiratory diseases. These conditions are endured by millions of people worldwide and represent a significant cause of morbidity and mortality. Currently, there are no effective treatments for the majority of advanced lung diseases and lung transplantation remains the only hope for many chronically ill patients. Key opinion leaders speculate that the novel coronavirus, COVID-19, may lead to long-term lung damage, further exacerbating the need for regenerative therapies. New strategies for regenerative cell-based therapies harness the differentiation capability of human iPSCs for studying pulmonary disease pathogenesis and treatment. Excitingly, biomaterials are a cell culture platform that can be precisely designed to direct stem cell differentiation. Here, we present a closer look at the state-of-the-art of iPSC differentiation for pulmonary engineering, offer evidence supporting the power of biomaterials to improve stem cell differentiation, and discuss our perspective on the potential for tissue-informed biomaterials to transform pulmonary regenerative medicine.

## Introduction

Chronic respiratory diseases are the third leading cause of global morbidity and mortality, impacting an astonishing 7.4% of the world's population (1). Despite progress in therapeutic development for these conditions, current treatments merely control symptoms and exacerbations. The urgency for new treatment options cannot be underestimated as escalating urban environmental risk factors and tobacco use, have caused a substantial increase in the exacerbation and mortality rates of chronic lower respiratory diseases (2). This growth is exemplified by the 39.8% increase chronic respiratory disease cases since 1990, which includes a 3.9% global increase in chronic obstructive pulmonary disease (COPD) and a 3.6% increase in asthma (2). Increased air pollution exposure also heightens the risk for exacerbations in COPD and idiopathic pulmonary fibrosis (IPF) (3, 4). Furthermore, researchers have projected that survivors of severe acute respiratory syndrome coronavirus 2 (SARS-CoV-2), responsible for the recent global pandemic, will have an increased prevalence of chronic respiratory conditions due to severe lung damage caused by acute respiratory distress syndrome (ARDS) (5). Collectively, airway damage and scarring of gas-exchange surfaces in the lungs will require innovative therapeutic strategies for airway regeneration. Nevertheless, progression of new therapeutic approaches for these diseases is hindered by a lack of reproducible *in vitro* models that closely reflect *in vivo* physiology.

**Figure 1:**
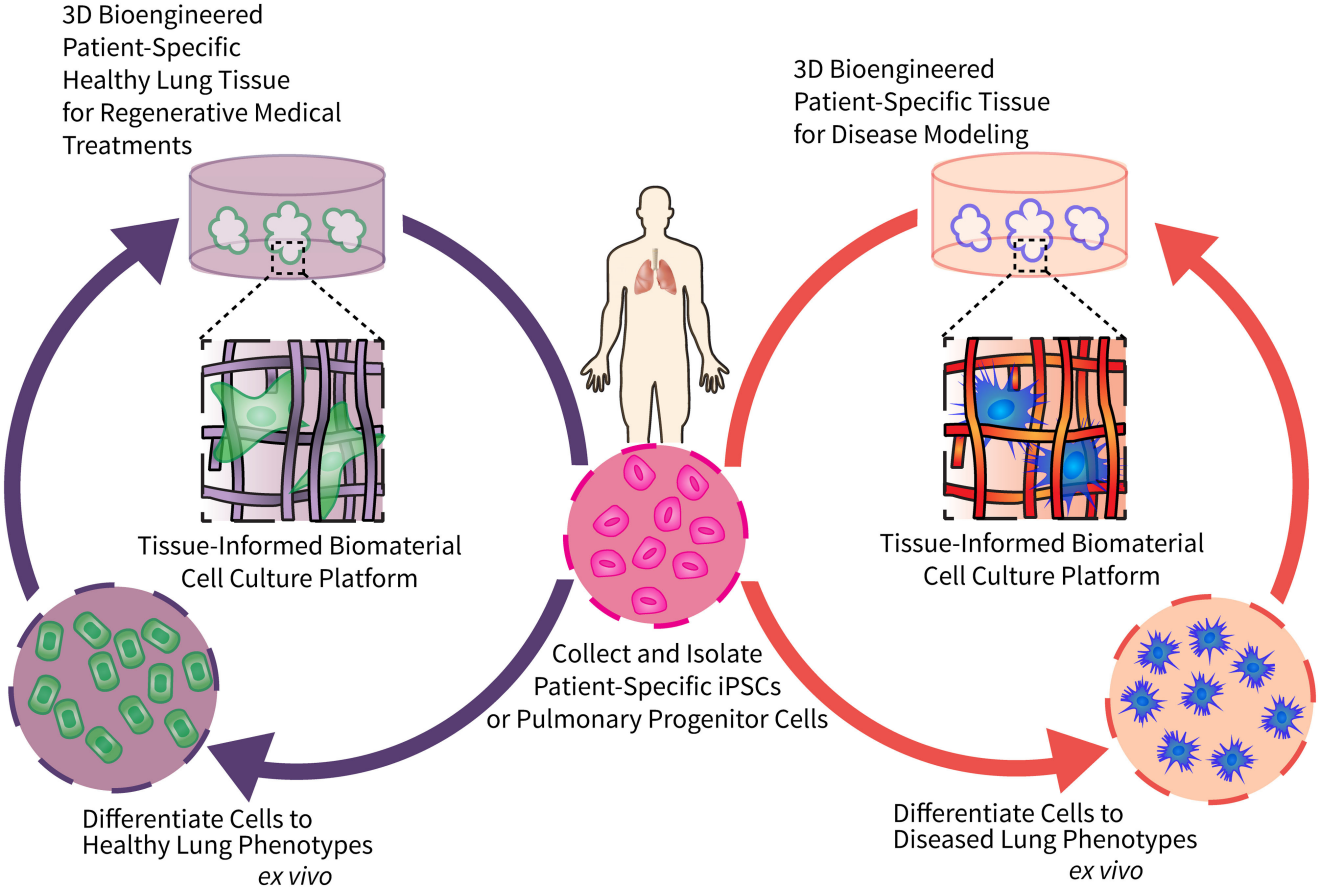
Engineering tissue-informed biomaterials to advance pulmonary regenerative medicine and model respiratory disease. This schematic illustrates the potential for tissue-informed biomaterials to advance pulmonary regenerative medicine through two complementary pathways: (1) by using tissue-informed biomaterial cell culture platforms to differentiate patient-specific stem cells into healthy, implantable lung cells and tissue (left); and (2) engineering biomaterial-based platforms to induce differentiation of these same patient-specific stem cells into diseased phenotypes for disease modeling and evaluation of precision medical treatments (right).

Respiratory diseases present with significant heterogeneity among patients, creating considerable variability in disease onset, severity, and progression (6). Patient-specific regenerative cell-based therapies provide an attractive avenue to study pulmonary disease pathology and evaluate effective treatment regimes. However, progress is currently limited by materials that are not conducive to optimization through controlled modification. To support the complex cell culture processes required to build these models, we present here the perspective that precisely engineered biomaterials will increase the reproducibility and efficiency of patient-derived induced pluripotent stem cells (iPSCs) differentiation, facilitating the fabrication of three-dimensional (3D), patient-specific models of pulmonary regeneration and disease. Figure 1 depicts a long-term vision for how tissue-informed biomaterials can improve pulmonary regenerative medicine and discovery of new therapeutic targets. First, patient-specific cells can be isolated, expanded, reprogrammed into pluripotent stem cells, and differentiated into mature lung cells using engineered biomaterial cell culture platforms. Biomaterial microenvironments can be tailored to differentiate cells to either healthy lung cells/tissues for transplantation, or diseased lung phenotypes for modeling and evaluating the impact of treatments on chronic respiratory conditions. We predict that this versatility will enable researchers to tissue-informed biomaterials and patient-derived cells to address the unique challenges of each individual respiratory disease.

## Current Strategies for Modeling Human Lung Development and Regeneration

### Animal Models for Studying Respiratory Disease

Animal models are widely used to study lung development and disease pathology. Murine models, for example, have been fundamental in examining physiological processes such as branching morphogenesis, which develops lung architecture and tubular structures (7). Nevertheless, there are discrepancies between rodent and human lung development, physiology, and pathophysiology which have led to numerous successful preclinical animal therapeutic successes that later fail in human clinical trials (8). Murine models, although capable of developing some lung disease phenotypes, such as goblet cell hyperplasia and airway mucus obstruction in COPD, do not develop spontaneous bacterial infections or have equivalent levels of disease severity (9). One example of the limitations of rodent models are those created to study cystic fibrosis (CF), a respiratory disease caused by mutations within the cystic fibrosis transmembrane conductance regulator (CFTR) gene, resulting in the inability to effectively transport chloride across the cell membrane and the accumulation of thick mucus that obstructs airways. In these models, mice generally present a less-severe lung phenotype due to activity of alternative chloride channels that can mitigate the burden of mutated CFTR (10). A transition to large-animal model systems, specifically ferrets and pigs, has improved recapitulation of human lung disease (11, 12). Pigs and ferrets more closely resemble human lung anatomy and morphology, with airways that exhibit similar bacteria and immune cell infiltration (13, 14). Moreover, they are capable of developing spontaneous lung disease and similar lung pathologies such as airway obstruction, inflammation, and mucus buildup to humans (12, 14). While these models have been beneficial in studying disease onset and pathogenesis, they are costly and not ideal for high-throughput therapeutic studies or screening.

### Patient-Derived Cells for Studying Respiratory Disease

Patient-derived primary lung epithelial cells have been used as an *in vitro* alternative to animal models due to their ability to replicate *in vivo* cell morphology, physiology, and functionality (15, 16). Significant advances have been made in the development of procedures for procuring lung tissue explants and bronchoscopy samples, isolating human airway epithelial cells (HAECs), and optimizing media and culture conditions for their expansion. The cell types that comprise the airway epithelium are summarized in Table 1. Protocols, such as those developed by (32), support the growth and mucociliary differentiation of HAECs at the air-liquid interface (ALI) (33). Similar methods have grown patient primary epithelial cells for therapeutic testing. For example, Neuberger et al., isolated human bronchial epithelial cells (HBECs) from CF patients to perform preclinical tests for CFTR modulators (15). Nevertheless, there are many challenges to working with primary HAECs, including limited access to patient samples, particularly those with rare diseases and genotypes. Furthermore, *in vitro* expansion of primary cells is limited due to decreased proliferation over time, changes in morphology, and loss of multipotency of the predominant airway epithelium progenitor cell, the basal cell, which has the capacity to regenerate the airway epithelium of the trachea to the bronchioles (34, 35).

**Table 1:** Summary of human airway structure, cellular composition, cellular characteristics, differentiation potential, and derivation from embryonic stem cells (ESC) and induced pluripotent stem cells (iPSC).

**Human airway structure**	**Cell type**	**Function**	**Differentiation potential**	**Isolation/markers**	**Putative derivation from ESC/iPSC**	**References**
Upper airway	Basal	Airway progenitors/junctional adhesion/inflammatory response	Airway epithelium	Bronchial brushing, pronase digestion/TRP63, KRT5, NGFR+, Pdpn	Yes	(17–22)
	Club	Progenitor cells/ secrete surfactants	Ciliated, goblet, AT1, and AT2	Basal cell differentiation/Scgb1a1	Yes	(17, 22–25)
	goblet	Mucus production and secretion	None	Basal cell differentiation/MUC5AC	Yes	(17, 22, 26)
	Ciliated	Mucociliary clearance	None	Basal cell differentiation/FOXJ1, acetylated tubulin	Yes	(17, 22, 27, 28)
Lower airway	AT1	Gas exchange/ion and fluid transport	None	Elastase digestion, magnetic sorting, FACS/RTI- 40	Yes	(17, 29)
	AT2	Alveolar stem cells/secrete pulmonary surfactant/ ion transport	AT1	Elastase digestion, magnetic sorting, FACS/RTII- 70	Yes	(17, 29–31)

The invention of iPSCs in 2007 created an alternative approach to obtaining patient-specific cells capable of self-renewal, large-scale expansion, and multilineage differentiation (36–38). Investigation of effective methods to differentiate human iPSCs into mature airway epithelium has been crucial to developing functional cells for effective disease modeling and therapeutic screening. iPSCs have been directed to mimic embryonic lung development and differentiate into functional airway epithelium (17, 18, 27, 39, 40) through sequential addition of factors that regulate activin/nodal, bone morphogenic protein (BMP), fibroblast growth factor (FGF), transforming growth factor-beta (TGFβ), wingless (Wnt) and sonic hedgehog (Shh) signaling (Table 1). These strategic methods of differentiation to lung cell lineages have primarily focused on two-dimensional (2D) monolayer cultures and have varying levels of efficiency based on induction of NK2 homeobox 1 (NKX2.1) expressing primordial lung progenitor cells. Currently, 2D systems are not capable of proper spatial tissue organization and epithelial-mesenchymal associations (41).

Cell culture substrates with physiological mechanical properties and 3D architecture may improve differentiation efficiency and the formation of mature cells from stem or progenitor cells that are more similar to their primary cell counterparts in gene expression and DNA methylation profiles, with studies having largely focused on natural materials, such as basement membrane extracts (41), decellularized precision-cut lung slices (42), and hydrogels derived from extracellular matrix (ECM) (43) to differentiate human lung progenitor cells and evaluate the resulting cellular structure and function. A summary of lung ECM components, cellular binding regions, and common strategies for incorporating these biochemical cues into biomaterials can be found in Table 2.

**Table 2:** Introduction to common ECM components found in the lung, cell binding sites, and common practices for incorporation into biomaterials.

**ECM macromolecule**	**Cell binding site**	**Representative peptide sequence or component for incorporation into biomaterials**	**Function in lungs**	**References**
**Collagens**				
Col I and III Col IV	α_1_β_1_, α_2_β_1_, α_3_β_1_ α_1_β_1_, α_2_β_1_	gly-phe-hyp-gly-glu-arg (GFOGER) glu-phe-tyr-phe-asp-leu-arg-leu-lys-gly-asp-lys (EFYFDLRLKGDK)	Central airways, Alveolar ducts, and Interstitium of the parenchyma Basement membrane	(44–48)
**Elastic fibers**				
Elastin, Fibrillin-1, Fibrillin-2, and Fibulin-5 Elastin microfibril interface-located proteins (EMILINs)	α_V_β_3_ α_V_β_3_, α_5_β_1_	val-ala-pro-gly (VAPG) arg-gly-asp (RGD)	Interstitium of the parenchyma	(44, 45, 49–52)
Laminins	α_3_β_1_, α_5_β_1_, α_6_β_4_	tyr-lle-gly-ser-arg (YIGSR) lle-lys-val-ala-val (IKVAV)	Basement membrane	(47, 53, 54)
Fibronectin	α_V_β_3_, α_5_β_1_	arg-gly-asp (RGD)	Basement membrane	(45, 47, 52–56)
Glycosaminoglycans (GAGs) Heparin/Heparan Sulfate Hyaluronic Acid Chondroitin Sulfate Dermatan Sulfate Keratan Sulfate	Non-integrin binding	GAGs can be incorporated into hybrid-hydrogels or used as coatings	Interstitium of the parenchyma	(44, 56, 57)

An investigation by Young et al. compared the epithelial barrier function of human basal epithelial stem cells cultured on combinations of a variety of proteins, including collagen I, fibronectin, laminin, and decellularized extracellular matrix (dECM). The combination that produced the highest barrier function as measured by trans-epithelial electrical resistance (TEER) was dECM supplemented with laminin (58). In another study by Greaney et al. human basal progenitor cells were seeded and epithelial differentiation was compared on various platforms including sections of decellularized lung tissue from trachea and distal lung, Matrigel, and traditional ALI culture (59). The study found that cells seeded on sections of decellularized lung tissue sections exhibited regionally specific indicators of epithelial regeneration such as detection of relevant airway epithelial cell markers cytokeratin 5 (KRT5, basal cells), mucin 5AC (MUC5AC, goblet cells), and acetylated alpha tubulin (ATUB, ciliated cells) on decellularized tracheal sections using single cell RNA sequencing (scRNA-seq). These 3D culture systems outperformed 2D culture on basement membrane extracts (Matrigel) and traditional ALI culture. Another recent study investigated seeding of primary human epithelial progenitor cells into hybrid bioinks composed of alginate reinforced with dECM. This hybrid approach allowed for the creation of bioinks with higher viscosities at low shear rates when compared to normal alginate. Epithelial progenitor cells were seeded into this hybrid bioink and 3D printed as a hollow tube and then subjected to ALI differentiation for 28 days. The study found that the hybrid bioink allowed for the differentiation of progenitor cells into ATUB-expressing ciliated cells and that the constructs remained stable and patent for the full 28 days (60). In contrast, culturing human lung organoids (HLOs) derived from pluripotent stem cells within Matrigel enabled cells to spontaneously form physiologically elaborate 3D structures *in vitro*. These cultures were composed of both epithelial and mesenchymal lineages that corresponded to the cellular composition and structural attributes of the human fetal lung. The approach by Dye et al. produced 3D structures resembling bronchi and bronchioles using HLOs derived from human pluripotent stem cells. However, in a comparable study carried out by Goetzke et al., significant differences in the epigenetic regulation of the cells were observed in iPSCs differentiated on 2D fibrin-based hydrogels compared to 3D culture in the same material. In fact, iPSCs differentiated into induced mesenchymal stem cells (iMSCs) in the 2D condition and were a closer match to primary MSC than the 3D differentiated cells. The cells differentiated in 3D had a notable upregulation of genes related to the cardiovascular system and neurogenesis (61). These results indicate that even though 3D systems are more physiologically relevant and represent an increased complexity compared to 2D systems, these platforms still need further optimization to be more physiologically precise.

### Limitations of Current Substrates for iPSC Differentiation

Currently, iPSC differentiation protocols rely extensively on natural, xenogenic materials such as Geltrex and Matrigel, which are basement membrane extracts rich in laminin-111, collagen IV, entactin, and perlecan (55). These extracts act as a substrate for cellular adhesion and present biological moieties that influence cell growth and differentiation at an epigenetic level (62). Unfortunately, these materials also exhibit major translational limitations. Geltrex and Matrigel are both derived from murine tumor tissue, resulting in a high potential for immunogenicity and poorly defined composition with reports of batch-to-batch variability from the manufacturers (63). This heterogeneity contributes to lower differentiation efficiency and limits scalability in future drug development work. Most importantly, these materials lack the capacity for tunability, or customization, required to optimize the substrates for reproducible and efficient iPSC to lung progenitor cell differentiation (64). Synthetic biomaterials designed using tissue-informed engineering strategies can overcome the limitations of traditional materials such as Matrigel and increase the efficiency of differentiating iPSCs into mature cells for further study (30, 65). Specifically, the following section highlights the implementation of tissue-informed hydrogels to support iPSC differentiation protocols for pulmonary regenerative medicine.

## Opportunities for Tissue-Informed Biomaterials to Advance Pulmonary Regenerative Medicine

Tissue-informed engineering strategies are a bottom-up approach to engineering materials meant to elicit specific cellular responses (66). First, key facets of tissues are characterized, including the extracellular matrix structure, mechanics, and composition. Next, the intrinsic and extrinsic properties of biomaterials are specifically engineered to replicate the tissues that support cellular expansion, differentiation, and maintenance within a 3D tissue-like architecture. Hydrogels, such as poly(ethylene glycol) PEG, have emerged as a promising candidate for the tissue-informed engineering process. Hydrogels are a single molecule network composed of cross-linked polymer chains. This cross-linking confers an advantage to hydrogels: the ability to swell in water without dissolving allows these materials to closely mimic the mechanical properties and water content of human tissue. Hydrogels have intrinsic and extrinsic properties that can be engineered and optimized using an iterative design process to achieve cellular responses appropriate for each application (67, 68). Intrinsic properties include stiffness (elastic modulus), degradability, and viscoelasticity. Extrinsic properties include dimensionality, topography, and presentation of biomolecules (69).

Tuning the intrinsic and extrinsic properties of these materials takes various forms, ranging from optimizing a static 2D hydrogel cell culture substrate to designing a stimuli-responsive, 3D material system that can be altered by user-controlled inputs or endogenous signals from embedded cells (70, 71). The elastic modulus (E) or stiffness of the hydrogel microenvironment is one physical (intrinsic) property that has been modified by exploiting user-controlled stimuli including light, temperature, or even ultrasound (71). A photodegradable PEG-based crosslinker developed by the Anseth research group, for instance, facilitated dynamic hydrogel softening from E > 30 kPa to E <3 kPa upon exposure to ultraviolet (UV) light (72, 73). This material was used to study gastrointestinal crypt formation by iPSCs embedded in 3D PEG hydrogels compared to 3D Matrigel constructs. The study found that crypt formation, size, and number per colony were functions of matrix softening. It also showed that colony survival was dependent on elastic modulus, with the greatest survival occurring in matrices with a modulus of 1.3 kPa (74). Similarly, one extrinsic property to leverage when designing synthetic materials is the incorporation and release of biological molecules with spatial and temporal control. These moieties include but are not limited to growth factors (75), peptides (76), protein fragments (77), and small molecules (78). For example, Ovadia et al. fabricated PEG-norbornene hydrogel matrices crosslinked with a cell-degradable peptide that presented pendant peptides inspired by proteins and integrins found in Matrigel to cells grown within these constructs. This research found that certain peptide combinations, specifically YIGSR (mimicking laminin) and PHSRNG_10_RGDS (replicating fibronectin-binding sites) enhanced viability of iPSCs and allowed for differentiation into neural progenitor cells (NPCs) when cultured in 3D over 1 week (79). Another study by Lam et al. used a design of experiments approach to optimize peptide concentrations in engineered biomaterials to maximize the differentiation of iPSCs to NPCs (67). These examples highlight significant progress in the differentiation of iPSCs using biomaterials for non-pulmonary engineering applications.

Tuning the intrinsic and extrinsic properties of biomaterials using a tissue-informed approach has the potential to pave the way for a transformation in pulmonary regenerative medicine. Although there are currently fewer examples, engineered biomaterials have been incorporated into pulmonary medical research platforms for tissue regeneration and disease modeling. Bailey et al. systematically evaluated PEG-based hydrogels for supporting extended *ex vivo* culture of precision-cut lung slices, specifically the maintenance of alveolar epithelial type II (ATII) cells, a progenitor cell capable of differentiation into alveolar epithelial type I (ATI) cells, the cells lining the gas exchange surfaces of the lung. This research demonstrated that incorporation of two short peptide sequences that bind β_1_-class integrins (0.2 mM YIGSR and 0.1 mM RGDS) supported production of surfactant protein c, that is, maintained the functionality of ATII cells within PLCS for up to 21 days, in contrast to unembedded controls that only survive ~7 days in culture (53). In a recent example of pulmonary disease modeling, a dynamically responsive PEG-α-methacrylate (PEGαMA) hybrid-hydrogel containing proteins from decellularized lung extracellular matrix was stiffened *in situ* using light to increase the elastic modulus of the material from healthy (E = 3.6 ± 0.24 kPa) to fibrotic ranges (E = 13.4 ± 0.82 kPa). These stiffened hydrogels induced a significant increase in the expression of myofibroblast transgenes within primary murine fibroblasts (80). Likewise, Lewis et al. exploited photodegradable PEG-based hydrogel microspheres to template lung epithelial cells within a biomaterial platform to create open cyst-like structures (81). These 3D model systems were used to demonstrate that fibroblasts in the surrounding hydrogel matrix responded to changes in epithelial cell activity by increasing proliferation and migration when co-cultured with a human tumor-derived epithelial cell line (A549) (82).

It is exciting to imagine a future where tissue-informed biomaterials can incorporate and release biomolecules to sequentially guide stem cell differentiation pathways such as integrin-binding peptides, cytokines, or small molecules (79, 83). This tunability of intrinsic material properties could enable more efficient patient stem cell differentiation toward mature lung tissue. During the progression of many chronic respiratory diseases, considerable changes to the mechanical properties of the lung tissue have been characterized (66). In fibrotic diseases such as idiopathic pulmonary fibrosis and pulmonary arterial hypertension, an aberrant healing response and excess collagen deposition lead to increases in lung stiffness from 1–5 kPa (healthy) to over 10 kPa (fibrotic) (84, 85), while COPD results in an overall decrease in tissue organization and stiffness (85). Biomaterials mimicking these dynamic changes in extracellular matrix mechanics could be readily designed to provide sophisticated *in vitro* models of patient-specific disease and treatment (80, 86). Currently, the strength of tissue-informed biomaterials has not been harnessed in pulmonary medicine, but the opportunities are substantial and should continue to be investigated in the future.

## Outlook

The number of persons affected by chronic respiratory disease worldwide has grown significantly in the last three decades. The COVID-19 pandemic has provided clinical data showing pulmonary fibrosis in those that survive the infection (5, 87). It is hypothesized that this fibrotic response will not regress, leading to a latent burgeoning of chronic respiratory disease in the future. To improve quality of life in patients with chronic diseases, we must understand the disease so that we may engineer the proper treatments. As of now, lung transplantation is the only effective treatment for patients with severe chronic respiratory disease, and the current need far outweighs the available supply. We envision that the solution sits at the intersection of patient-derived stem cells and tissue-informed biomaterials. By engineering biomaterials that can mimic human tissue, we can guide patient stem cells in differentiation toward regeneration of healthy lung tissue or disease models for studying precision medical treatments.

## Data Availability Statement

The original contributions presented in the study are included in the article/supplementary material, further inquiries can be directed to the corresponding author/s.

## Author Contributions

DC, CS, AR, and CM worked together to conceptualize the content of this perspective. CS and AR wrote the current strategies for modeling human lung development and regeneration section. DC and CM wrote the Opportunities for Tissue-Informed Biomaterials to Advance Pulmonary Regenerative Medicine section, and conceptualized and designed Figure 1. AR and CM shared responsibility for writing and editing the Introduction and Outlook. CM oversaw final edits of the manuscript. All authors contributed extensively to the work presented in this manuscript.

## Conflict of Interest

The authors declare that the research was conducted in the absence of any commercial or financial relationships that could be construed as a potential conflict of interest.
